# Supplementary material for: External Validation of Prediction Models for Surgical Complications in People Considering Total Hip or Knee Arthroplasty Was Successful for Delirium but Not for Surgical Site Infection, Postoperative Bleeding, and Nerve Damage: A Retrospective Cohort Study
Source: J Pers Med. 2023 Jan 31;13(2):277. doi: 10.3390/jpm13020277 (PMC9964485; doi:10.3390/jpm13020277)
Supplement: Supplementary file 1 [file jpm-13-00277-s001.zip › jpm-2097353-supplementary.pdf]

Supplementary Materials

Supplementary Materials Table S1.

Supplementary Materials Figure S1.

Supplementary Materials Table S1. Query CTcue

Question	Filter
**Which procedure was performed? (THP, TKP or UKP)**	
THP<br><br><br><br>TKP<br><br><br><br>UKP	*Specialism*: Orthopaedics<br>*Start date*: ≥ 01-01-2017<br>*Age at time of event*: >12 years<br><br>*Specialism*: Orthopaedics<br>*Start date*: ≥ 01-01-2017<br>*Age at time of event*: >12 years<br><br>*Specialism*: Orthopaedics<br>*Start date*: ≥ 01-01-2017<br>*Age at time of event*: >12 years<br><br>*Used term*: surgical procedures, specified with procedure codes<br>*Result preferences*: oldest (ascending)
**What is the length, weight and BMI of the patient?**	
Length<br><br><br><br>Weight<br><br><br><br>BMI	*Description:* Length<br>*Start date:* before <THP, TKP, UKP<br>Include events that happened later on the same day<br><br>*Description:* Weight<br>*Start date:* before <THP, TKP, UKP<br>Include events that happened later on the same day<br><br>*Description:* BMI<br>*Start date:* before <THP, TKP, UKP<br>Include events that happened later on the same day<br><br>*Used term:* vital signs<br>*Result preferences*: newest (descending)
**Does the patient smoke?**	
Yes<br><br><br><br><br>No	*Description (reports):* smoking status: +, and search in context with specified texts<br>*Description (forms):* smoking, yes<br>*Start date:* before <THP, TKP, UKP<br>Include events that happened later on the same day<br><br>*Description (reports):* smoking status: -, and search in context with specified texts<br>*Description (forms):* stop smoking, never<br>*Start date:* before <THP, TKP, UKP<br>Include events that happened later on the same day<br><br>*Used term:* reports, forms<br>*Result preferences*: oldest (ascending)
**Diabetes mellitus**	
Yes	*Report type:* preoperative screening<br>*Description (reports):* diabetes mellitus<br>*Ingredient (medication administrations):* metformin, insulin, etcetera<br>*Start date:* before <THP, TKP, UKP<br>Include events that happened later on the same day<br><br>*Used term:* reports, medication administrations<br>*Result preferences*: newest (descending)
**Lung disease**	
Yes	*Report type:* preoperative screening, outpatient clinic first contact, outpatient clinic follow-up contact, outpatient clinic letter

	Description (reports): COPD, lung disease, asthma, etcetera<br>*Start date:* before <THP, TKP, UKP<br>Include events that happened later on the same day<br><br>*Used term:* reports<br>*Result preferences*: newest (descending)
**Rheumatoid arthritis**	
Yes	*Report type:* preoperative screening, outpatient clinic first contact, outpatient clinic follow-up contact, outpatient clinic letter<br>*Description (reports):* rheumatic diseases, rheumatism, rheumatics<br>*Start date:* before <THP, TKP, UKP<br>Include events that happened later on the same day<br><br>*Used term:* reports<br>*Result preferences:* newest (descending)
**Liver disease**	
Yes	*Report type:* preoperative screening, outpatient clinic first contact, outpatient clinic follow-up contact, outpatient clinic letter<br>*Description (reports):* fatty liver, liver cirrhosis, cirrhosis of liver, autoimmune hepatitis<br>*Start date:* before <THP, TKP, UKP<br>Include events that happened later on the same day<br><br>*Used term:* reports<br>*Result preferences:* newest (descending)
**Immunological disorder**	
Yes	*Report type:* preoperative screening, outpatient clinic first contact, outpatient clinic follow-up contact, outpatient clinic letter<br>*Description (reports):* scleroderma, psoriasis, systemic sclerosis, etcetera<br>*Start date:* before <THP, TKP, UKP<br><br>*Used term:* reports<br>*Result preferences:* newest (descending)
**Heart disease**	
Yes	*Report type:* preoperative screening, outpatient clinic first contact, outpatient clinic follow-up contact, outpatient clinic letter<br>*Description (reports):* Coronary Artery Bypass Grafting, heart attack, etcetera<br>*Start date:* before <THP, TKP, UKP<br><br>*Used term:* reports<br>*Result preferences:* newest (descending)<br>*Specialism:* anesthesiology, orthopedics
**Disease of the central nervous system**	
Yes	*Report type:* preoperative screening, outpatient clinic first contact, outpatient clinic follow-up contact, outpatient clinic letter<br>*Ingredient (medication administrations):* Levodopa, pramipexole, ropinirole<br>*Description (reports):* Parkinson disease, multiple sclerosis, huntington disease, etcetera<br>*Start date:* before <THP, TKP, UKP<br><br>*Used term:* reports<br>*Result preferences:* newest (descending)<br>*Specialism:* anesthesiology, orthopedics
**Previous thromboembolic event**	
Yes	*Report type:* preoperative screening, outpatient clinic first contact, outpatient clinic follow-up contact, outpatient clinic letter<br>*Description (reports):* (recurrent) deep vein thrombosis, pulmonary embolism<br>*Start date:* before <THP, TKP, UKP

	Used term: reports<br>*Result preferences:* newest (descending)<br>*Specialism:* anesthesiology
**Hip dysplasia**	
Yes	*Report type:* preoperative screening, outpatient clinic first contact, outpatient clinic follow-up contact, outpatient clinic letter<br>*Description (reports):* (congenital) hip dysplasia<br>*Start date:* before <THP, TKP, UKP<br><br>*Used term:* reports<br>*Result preferences:* newest (descending)<br>*Specialism:* anesthesiology, orthopedics
**Use of vitamin K antagonist**	
Yes	*Content (reports):* acenocoumarol, fenprocoumon<br>*Ingredient (medication administrations):* acenocoumarol, fenprocoumon<br>*Start date:* before <THP, TKP, UKP, AND <365 days before surgery<br>Include events that happened later on the same day<br><br>*Used term:* reports, medication administrations<br>*Result preferences*: newest (descending)
**Use of NSAIDs**	
Yes	*Content (reports):* NSAIDs, coxibs<br>*Ingredient:* indometacine, meloxicam, naproxen, etcetera<br>*Start date:* before <THP, TKP, UKP, AND <365 days before surgery<br>Include events that happened later on the same day<br><br>*Used term:* reports, medication administrations<br>*Result preferences*: newest (descending)
**Outcomes**	
**Surgical site infection**	
Yes	*Report type:* no specification<br>*Description (reports):* surgical site infection, etcetera<br>*Ingredient (medication administrations):* rifampicin<br>*Start date:* between surgery date and 90 days after surgery date<br><br>*Used term:* reports, medication administrations<br>*Result preferences:* newest (descending)
**Venous Thromboembolism**	
Yes	*Report type:* no specification<br>*Description (reports):* (recurrent) deep vein thrombosis, pulmonary embolism<br>*Start date:* between surgery date and 90 days after surgery date<br><br>*Used term:* reports<br>*Result preferences:* newest (descending)
**Postoperative bleeding**	
Yes	*Report type:* no specification<br>*Description (reports):* wound leakage, serosanguinal leakage, bleeding, etcetera<br>*Start date:* between surgery date and 90 days after surgery date<br><br>*Used term:* reports<br>*Result preferences:* newest (descending)
**Delirium**	
Yes	*Report type:* no specification<br>*Description (reports):* delirium<br>*Start date:* between surgery date and 90 days after surgery date<br><br>*Used term:* reports

	Result preferences: newest (descending)
**Nerve damage**	
Yes	*Report type:* no specification<br>*Description (reports):* nerve damage, nerve damage N. femoralis, N. ischiadicus, N. peroneus<br>*Start date:* between surgery date and 90 days after surgery date<br><br>*Used term:* reports<br>*Result preferences:* newest (descending)

Supplementary Materials Figure S1. Calibration plot per surgical complication indicating the calibration of the models

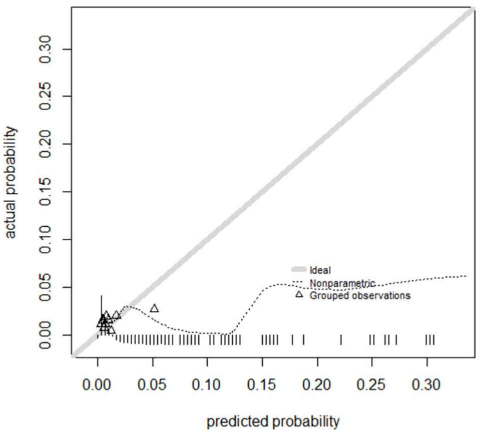

a. Surgical site infection

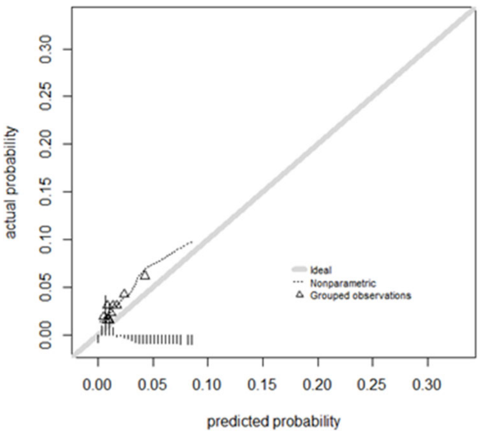

b. Postoperative bleeding

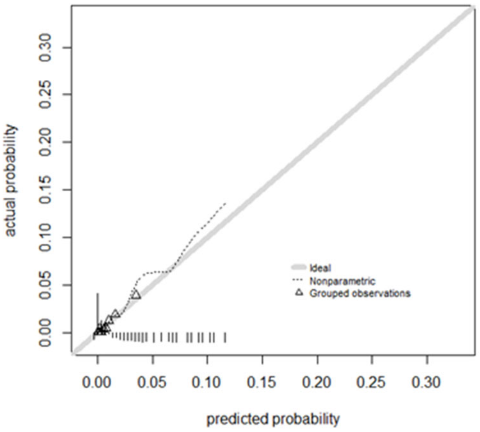

c. Delirium

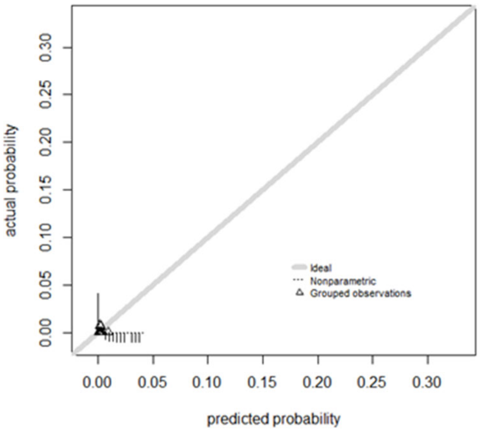

d. Nerve damage
